# Honey bees and climate explain viral prevalence in wild bee communities on a continental scale

**DOI:** 10.1038/s41598-022-05603-2

**Published:** 2022-02-03

**Authors:** Niels Piot, Oliver Schweiger, Ivan Meeus, Orlando Yañez, Lars Straub, Laura Villamar-Bouza, Pilar De la Rúa, Laura Jara, Carlos Ruiz, Martin Malmstrøm, Sandra Mustafa, Anders Nielsen, Marika Mänd, Reet Karise, Ivana Tlak-Gajger, Erkay Özgör, Nevin Keskin, Virginie Diévart, Anne Dalmon, Anna Gajda, Peter Neumann, Guy Smagghe, Peter Graystock, Rita Radzevičiūtė, Robert J. Paxton, Joachim R. de Miranda

**Affiliations:** 1 Department of Plants and Crops, University of Gent, Coupure Links 653, 9000 Gent, Belgium; 2 Department Community Ecology, UFZ-Helmholtz Centre for Environmental Research, Theodor Lieser Str. 4, Halle, Germany; 3 iDiv, German Centre for Integrative Biodiversity Research, Halle-Jena-Leipzig, Leipzig, Germany; 4 Institute of Bee Health, Vetsuisse Faculty, University of Bern, Bern, Switzerland; 5 Departamento de Zoología y Antropología Física, Facultad de Veterinaria, Universidad de Murcia, Campus de Espinardo, Murcia, Spain; 6 Department of Biosciences, Center for Ecological and Evolutionary Synthesis (CEES), University of Oslo, Oslo, Norway; 7 Department of Landscape and Biodiversity, Norwegian Institute of Bioeconomy Research (NIBIO), Ås, Norway; 8 Estonian University of Life Sciences, Kreutzwaldi 1, Tartu, Estonia; 9 Department for Biology and Pathology of Fish and Bees, Faculty of Veterinary Medicine University of Zagreb, Zagreb, Croatia; 10 Department of Molecular Biology and Genetics, Cyprus International University, Mersin 10, Nicosia, Turkey; 11 Department of Biology, Hacettepe University, Beytepe, Ankara, Turkey; 12 INRAE, Unité Abeilles et Environnement, Avignon, France; 13 Laboratory of Bee Diseases, Institute of Veterinary Medicine, Warsaw University of Life Sciences, Warsaw, Poland; 14 Department of Life Sciences, Imperial College London, Silwood Park Campus, Ascot, UK; 15 Molecular Evolution and Animal Systematics, Institute of Biology, University of Leipzig, Talstraβe 33, Leipzig, Germany; 16 Institute for Biology, Martin Luther University Halle-Wittenberg, 06120 Halle (Saale), Germany; 17 Department of Ecology, Swedish University of Agricultural Sciences, 750 07 Uppsala, Sweden; 18 European Food Safety Authority (EFSA), Parma, Italy; 19 Departamento de Biología Animal, Edafología y Geología, Facultad de Ciencias, Universidad de La Laguna, La Laguna, Tenerife Spain; 20 Department of Zoology and Entomology, University of Pretoria, Pretoria, 0002 South Africa

**Keywords:** Virus-host interactions, Viral epidemiology

## Abstract

Viruses are omnipresent, yet the knowledge on drivers of viral prevalence in wild host populations is often limited. Biotic factors, such as sympatric managed host species, as well as abiotic factors, such as climatic variables, are likely to impact viral prevalence. Managed and wild bees, which harbor several multi-host viruses with a mostly fecal–oral between-species transmission route, provide an excellent system with which to test for the impact of biotic and abiotic factors on viral prevalence in wild host populations. Here we show on a continental scale that the prevalence of three broad host viruses: the AKI-complex (*Acute bee paralysis virus*, *Kashmir bee virus* and *Israeli acute paralysis virus*), *Deformed wing virus*, and *Slow bee paralysis virus* in wild bee populations (bumble bees and solitary bees) is positively related to viral prevalence of sympatric honey bees as well as being impacted by climatic variables. The former highlights the need for good beekeeping practices, including *Varroa destructor* management to reduce honey bee viral infection and hive placement. Furthermore, we found that viral prevalence in wild bees is at its lowest at the extreme ends of both temperature and precipitation ranges. Under predicted climate change, the frequency of extremes in precipitation and temperature will continue to increase and may hence impact viral prevalence in wild bee communities.

## Introduction

Even though the presence of viral pathogens often goes unnoticed, they form an indispensable facet of ecosystems^1–3^. However, when the natural dynamic interactions between hosts and their viral pathogens are disturbed they can have devastating effects on their hosts, often modulated by host shifts, as recently demonstrated by the SARS-CoV-2 pandemic^4^. The interactions between viruses and their hosts are a complex and can be affected by biotic factors, such as contact between wild and domesticated animals or humans^5,6^, as well as abiotic factors, such as temperature or precipitation^7–13^. Hence, to fully understand what drives viral prevalence and what triggers its negative effects on host populations, a thorough understanding of the role of biotic and abiotic factors is crucial for both wildlife as well as human welfare.

The virosphere of bees is very diverse, yet most knowledge on bee viruses and pathogens in general originates from studies of the managed Western honey bee, *Apis mellifera*^14^. Data for managed honey bees show that the currently documented viruses appear to have a global distribution^14,15^. In this managed species the viral landscape is highly impacted by the presence of the ectoparasitic mite, *Varroa destructor*, an effective vector of several viruses found in honey bees^16,17^. In the last decade, sparked by reported global declines of wild bees, research focus has shifted to viruses in wild bees^18–20^. As is true for the majority of pathogens^21,22^, most viruses in bees can be detected in multiple hosts^16,23,24^. Furthermore, co-infections with multiple pathogens are common, where different pathogens can impact one another inside the host^20,25–28^. Moreover, the bee microbiome may modulate within host pathogen dynamics^29–31^. Various studies have also highlighted the impact of agrochemical exposure of bees on their interaction with pathogens (reviewed in^32^).

One omnipresent factor impacting the whole bee community and its interactions with pathogens is climate. Albeit, several studies having shown that climatic variables can impact both the honey bee as a host as well as the interactions with its (viral) pathogens^33–38^, these studies are restricted to local scales and no study has investigated these impacts at large geographical scales. To understand the impact of viral pathogens on bees, we need to understand that a multitude of pathogens interact with different bee species within the pollinator community and how these interactions are influenced by climatic conditions across large spatial scales. The influence of climatic conditions can either be direct, affecting a host’s lifestyle, or indirect, by affecting pathogen transmission. Since most bee pathogens are transmitted via the fecal–oral route, enabling inter- and intra-species transmission via shared flowers^16,39–42^, climatic variables, such as UV-exposure, temperature and precipitation could potentially influence pathogen survival on flowers and thereby their transmission^40^. Furthermore, climatic variables affect vegetation phenology^43^, flower attractiveness^44^ and diversity^45^, which may alter the transmission network via flowers^23^, as well as quality, quantity of floral resources^45–47^, which may impact host immunity^48,49^ and consequently pathogen susceptibility and transmission.

**Figure 1:**
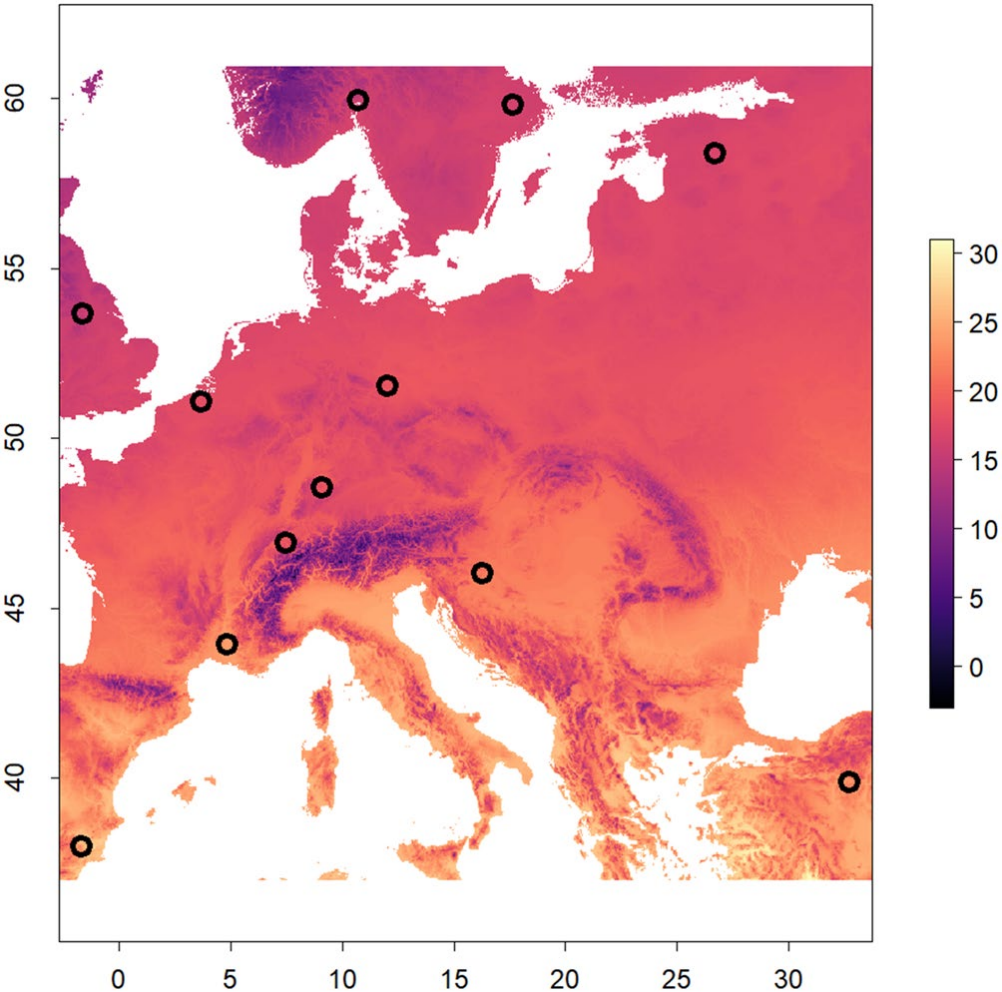
Map with the geographical distribution of the 12 sample sites (indicated with black circles) across Europe with color representing the climatic gradients (mean temperature of the warmest month [°C], as an example). Map created using R (version 4.0.4)^51^.

Here, we performed a pan-European assessment of the virosphere of 12 bee communities, each consisting of three bee groups: bumble bees, solitary bee species and sympatric managed honey bees, on a continental scale and across different climatic zones (Fig. 1). Using an AICc-based multi-model inference approach^50^, we related viral prevalence, i.e. fraction of specimens with positive virus detection per bee group, in wild bees to that of managed honey bees, climatic conditions, i.e. temperature and precipitation, and vegetation phenology and tested for their interactions, particularly addressing the following two hypotheses:

*H1* Viral prevalence in wild bees is entirely determined by abiotic climatic conditions.

*H2* Viral prevalence in wild bees is related to both biotic factors, namely viral prevalence in sympatric managed honey bees, as well as abiotic climatic conditions.

## Results

**Figure 2:**
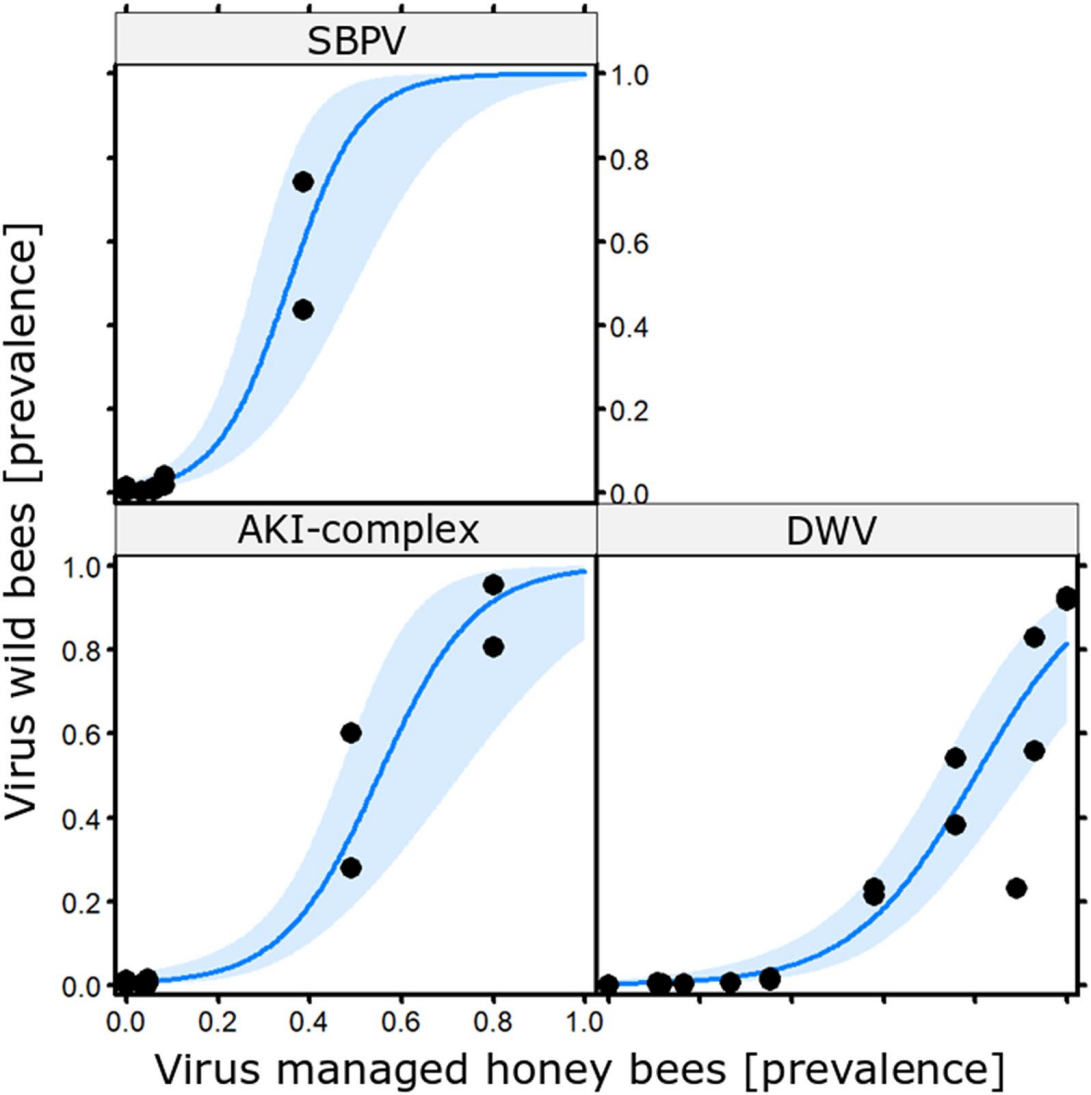
Relationship between viral prevalence in managed honey bees and wild bees split by virus. Analysis indicated a strong relation between the viral prevalence in honey bees and wild bees, the slope of the relation differs, however, between the viruses. For both SBPV (top panel) and the AKI-complex (bottom left panel) we see a clear increase in viral prevalence in wild bees as the viral prevalence in managed honey bees increases. For DWV (bottom right panel) we see a similar relation, yet only when the viral prevalence in honey bees increases above 50%. Shaded blue indicates the 95% confidential interval.

**Figure 3:**
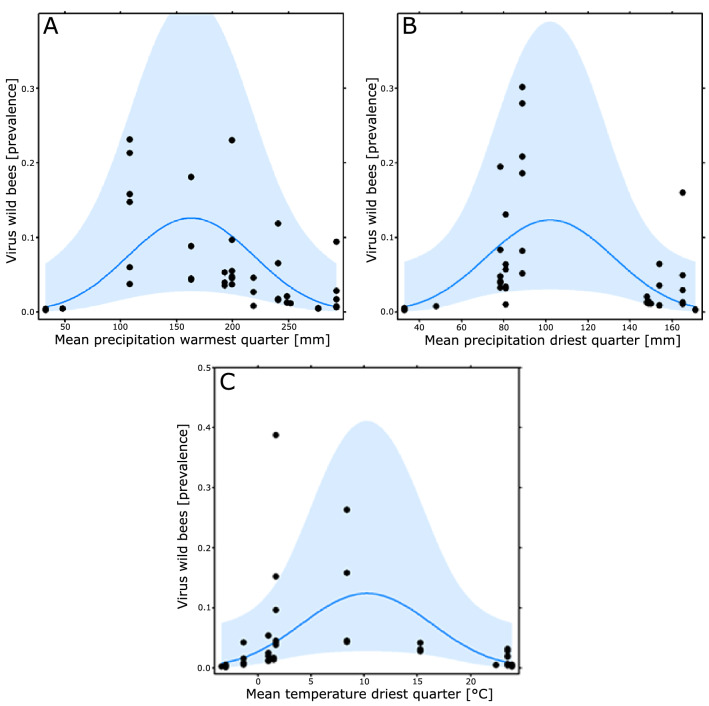
Relationship between viral prevalence (all viruses together) in wild bees (y- axis) and mean precipitation of the warmest quarter (**A**), mean precipitation of the driest quarter (**B**) and mean temperature of the driest quarter (**C**). Analysis indicated that the viral prevalence in wild bees was significantly affected by these three climatic variables. Precipitation and temperature means were obtained over a period of more than two decades at a resolution of 1–3 km. Shaded blue indicates the 95% confidential interval.

In total, we screened 1227 bee specimens (495 honey bees, 476 bumble bees, 256 solitary bees) for the following three virus species: *Deformed wing virus* (DWV), *Slow bee paralysis virus* (SBPV), AKI-virus complex (i.e. *Acute bee paralysis virus* (ABPV), *Kashmir bee virus* (KBV) and *Israeli acute paralysis virus* (IAPV)) and calculated viral prevalence for each of the three bee groups. Across Europe (see Fig. 1) and all three bee groups, DWV was the most prevalent virus, while all viruses showed considerable geographic variation in their prevalence (Supplementary Fig. 1). An exclusive impact of climatic conditions or length of the vegetation period (i.e., approximation for temporal extent of flower availability) on the observed geographic variation in virus prevalence (H1) was not supported. While virus prevalence in honey bees was not related to environmental conditions (see Supplementary Table 1), there was a strong positive relation with viral prevalence in wild bees (Table 1; column ‘*Apis’* present each model, with a positive coefficient), providing support for H2, i.e. a strong relationship between viral prevalence in honey bees and wild bees. The relationship with viral prevalence in honey bees was similar for both bumble bees and solitary bees (there was no interaction effect of species group). However, the relation was different for the three viruses (see Fig. 2). While the slope of this relationship was steep for the AKI-virus complex and SBPV, it was shallower for DWV, i.e., only higher prevalence of DWV in honey bees was reflected by an increase in DWV prevalence in wild bees (Fig. 2). In addition to the strong relation with viral prevalence in honey bees, wild bee viral prevalence depended also on climatic and vegetation conditions (Table 1), further supporting H2. The viral prevalence in wild bees generally declined across the year (Table 1; column ‘Phen’ indicated by a negative coefficient in all models). We also found virus species-specific responses to the temperature of the warmest quarter. This impact was negligible for DWV and SBPV, while the AKI-virus complex prevalence decreased with the temperature of the warmest quarter (Supplementary Fig. 3). The impact of precipitation of the warmest and driest quarter (Fig. 3A,B) and temperature of the driest quarter (Fig. 3C) was hump-shaped. Viral prevalence in wild bees was particularly low at both high and low levels of precipitation and temperature but without differences among the viruses.

**Table 1:** Multi-model inference results for separate analyses of climatic and vegetation phenological conditions ordered with increasing AICc.

Apis	Phen	Env	Env^2^	Virus	Apis:Env	Apis:Virus	Env:Virus	AICc	Delta	Weight	R^2^m	R^2^c
**Precipitation warmest quarters**												
1.29	− 0.78		− 1.04					228.94	0.00	0.65	0.33	0.67
1.30	− 0.84	0.15	− 0.98					231.60	2.66	0.17	0.35	0.67
1.45								232.84	3.90	0.09	0.27	0.58
1.53	− 0.41							232.85	3.92	0.09	0.31	0.59
**Temperature warmest quarter**												
2.52	− 0.55	− 1.40		+			+	230.89	0.00	0.18	0.51	0.64
1.34	− 0.75	− 0.92						231.83	0.94	0.11	0.26	0.66
2.68		− 1.16		+			+	232.15	1.27	0.10	0.53	0.66
2.78	− 0.56	− 1.22	− 0.34	+			+	232.64	1.76	0.07	0.56	0.66
1.45								232.84	1.95	0.07	0.27	0.58
1.53	− 0.41							232.85	1.97	0.07	0.31	0.59
2.38	− 0.58	− 1.57		+	0.30		+	233.00	2.11	0.06	0.52	0.64
3.00	− 0.48	− 1.55		+		+	+	233.17	2.28	0.06	0.56	0.66
3.10		− 1.52		+		+	+	233.32	2.43	0.05	0.55	0.66
1.36	− 0.73	− 0.91			0.17			233.59	2.71	0.05	0.28	0.65
2.68	− 0.57	− 1.40	− 0.52	+	0.43		+	233.83	2.95	0.04	0.58	0.67
2.71		− 1.06	− 0.25	+			+	234.32	3.44	0.03	0.54	0.66
1.33	− 0.79	− 0.91	− 0.16					234.39	3.50	0.03	0.26	0.67
2.44		− 1.25		+	0.25		+	234.55	3.66	0.03	0.51	0.64
2.16	− 0.48			+				234.67	3.79	0.03	0.42	0.63
1.37		− 0.27						234.73	3.84	0.03	0.23	0.59
**Precipitation driest quarter**												
1.36	− 0.54		− 1.02					231.02	0.00	0.30	0.33	0.63
1.34		− 0.71						232.72	1.71	0.13	0.28	0.59
1.45								232.84	1.82	0.12	0.27	0.58
1.53	− 0.41							232.85	1.84	0.12	0.31	0.59
1.40	− 0.68	0.24	− 0.90					233.46	2.45	0.09	0.36	0.62
2.32	− 0.89	0.60		+				233.65	2.63	0.08	0.50	0.66
1.60	− 0.73	0.47						233.89	2.87	0.07	0.37	0.61
1.29		− 0.32	− 0.98					234.35	3.33	0.06	0.27	0.62
2.16	− 0.48				+			234.67	3.66	0.05	0.42	0.63
**Temperature driest quarter**												
1.41	− 0.75		− 0.98					231.19	0.00	0.32	0.30	0.62
1.45								232.84	1.65	0.14	0.27	0.58
1.53	− 0.41							232.85	1.67	0.14	0.31	0.59
1.43	− 0.81	0.41	− 1.29					233.54	2.36	0.10	0.32	0.61
1.45	− 0.45	− 0.50						234.16	2.97	0.07	0.28	0.61
1.39			− 0.34					234.48	3.30	0.06	0.25	0.58
1.40		− 0.35						234.59	3.41	0.06	0.24	0.59
1.83	− 0.72		− 0.79	+				234.59	3.41	0.06	0.37	0.61
2.16	− 0.48			+				234.67	3.49	0.06	0.42	0.63
**Length of vegetation period**												
1.44	− 1.24	1.12						231.69	0.00	0.26	0.40	0.67
1.45								232.84	1.15	0.15	0.27	0.58
1.53	− 0.41							232.85	1.17	0.15	0.31	0.59
1.38	− 1.39	0.65	0.49					233.76	2.07	0.09	0.33	0.66
1.46	− 1.25	1.15			− 0.04			234.37	2.68	0.07	0.41	0.67
2.16	− 0.48			+				234.67	2.98	0.06	0.42	0.63
1.87	− 1.07	0.86		+				234.88	3.20	0.05	0.44	0.65
1.49			− 0.11					235.09	3.41	0.05	0.29	0.59
1.94	− 0.55			+		+		235.28	3.59	0.04	0.49	0.64
1.94				+				235.31	3.62	0.04	0.36	0.60
1.44		0.05						235.34	3.65	0.04	0.26	0.58

*Apis*, viral prevalence in *Apis mellifera*; Phen, vegetation phenology during sampling; Env, linear term of respective environmental variable; Env^2^, quadratic term of respective environmental variable; Virus, virus species; colon indicates interaction terms. Numbers are coefficient estimates of continuous variables; ‘+’ indicates relevance of categorical variables. AICc, Akaike information criterion corrected for small sample sizes; Delta; delta AICc; Weight, Akaike weight; R^2^m, marginal pseudo-R^2^ (only fixed effects^52^); R^2^c, conditional pseudo-R^2^ (fixed and random effects). Models with delta AICc < 2 indicate strong support and are marked with grey background. White background: models with delta AICc between 2 and 4, indicating weaker support.

The impacts of vegetation period or environmental conditions on the relation between viral prevalence in managed honey bees and wild bees were only weakly supported (2 < ΔAICc < 4; Table 1: column ‘Apis: Env). Of all environmental conditions, only temperature of the warmest quarter had an impact on the relation in viral prevalence between honey bees and wild bees (Supplementary Fig. 2a) Further, we found that the relation between honey bee viral prevalence and viral prevalence in wild bees was less steep in areas with shorter vegetation periods, while it increased with the length of the vegetation period (Supplementary Fig. 2b).

## Discussion

Within this study we specifically address viral prevalence in wild bees with respect to viral prevalence of sympatric honey bees as a biotic factor, as well as temperature and precipitation as climatic abiotic factors. We find that wild bee viral prevalence is affected by both the biotic factor as well as the abiotic factors, supporting our second hypothesis. We find only a weakly supported interaction effect between honey bee viral prevalence and the climatic factors on wild bee viral prevalence (see Table 1 column ‘Apis: Env’; the models with an interaction term all have ΔAICc > 2).

The viral prevalence of multi-host viruses in the bee community is, amongst others, determined by the presence and density of different host species. As bee hosts are all somehow connected through their shared floral resources and viral transmission can be multidirectional for multi-host viruses in the bee community, we cannot demonstrate the directionality of viral transmission in our study. Nevertheless, we find a strong link between the viral prevalence in wild bees and that of sympatric honey bees. This result confirms earlier studies that demonstrate that viral prevalence in wild bees is related to viral prevalence of sympatric honey bees^19,20,53^. While these former studies were restricted to local^53^ and national scales^19,20^, we expand these relationships to a continental scale.

Interestingly, the effects of managed honey bee viral prevalence on wild bee viral prevalence did not differ between bumble bees and solitary bees, despite their different lifestyles and social organization, thereby underlining the importance of interspecific pathogen transmission, e.g. via shared floral resources^16,39,42,54,55^. Even though some solitary bee species are dietary specialists, visiting flowers of only a few plant species, the presence of generalist species such as honey bees connect many species in one network module, increasing the likelihood of viral transmission to all bee species present in the same environment^56–60^. However, we found that the relation between viral prevalence in honey bees and wild bees differs between viruses. There is a tight relationship for both AKI-virus complex and SBPV, with increasing wild bee prevalence when honey bee prevalence increases. Yet, although a similar relation was found for DWV, this relation only becomes apparent when viral prevalence in honey bees was high (see Fig. 2).

In contrast to AKI-virus complex and SBPV, DWV is a virus which is predominantly found in honey bees^20,59,61^. This discrepancy in viral prevalence is also reflected in our results, where DWV is always more prevalent in honey bees at our different sampling sites compared to the other viruses, namely the AKI-virus complex and SBPV. Experimental infection assays have shown that all three viruses can infect both wild bees as well as honey bees^19,62–65^. However, in contrast to wild bees, honey bees face the additional pressure of the parasitic mite *Varroa destructor*, which besides its virus-vectoring capacity can also weaken colonies, increasing the impact of viral infections^66,67^. A recent study highlighted the role of *V. destructor* in the transmission dynamics of DWV^55^. Incorporation of within-honey bee colony transmission, mediated by *V. destructor* vectoring and bee-to-bee transfer into a transmission model could explain the higher DWV prevalence in managed honey bees compared to wild bumble bees observed in the field^55^. These results may also explain why we only observed DWV in wild bees when its prevalence in honey bees was high and the environmental viral load is likely to have been high.

Although both the AKI-virus complex and SBPV can also be vectored and amplified to lethal levels in honey bee colonies by *V. destructor*, these viruses are more commonly found in wild bees^20,59,61,68,69^, suggesting key differences in host susceptibility and competence between these three viruses. The strong relations found in our study between viral prevalence in honey bees and wild bees together with the results from previous studies^19,55^ highlight the need for good beekeeping practices, which include proactive varroa monitoring and control by beekeepers as well as appropriate hive placement, where the beekeeper takes into account the presence of endangered wild bees, to prevent managed honey bee colonies becoming sources of virus amplification and dissemination to wild bees^19,55,70^.

In spite of the high variation in viral prevalence in managed honey bees across Europe (Supplementary Fig. 1), we found no relation between viral prevalence in managed honey bees and the investigated environmental conditions, temperature and precipitation (Supplementary Table 1). In contrast to most wild bee species, honey bees are highly social insects, who actively control the climate within the hive^71^, and can maintain large food reserves to overcome poor foraging conditions; these traits may make their viral dynamics independent from environmental conditions. Moreover, the active management of honey bees may contribute to the absence of an environmental effect on viral prevalence. Beekeeping practices, such as hive placement and water provisioning as well as varroa mite control and nutritional support^72^, could have a large impact on the health status and viral prevalence in colonies, hence they may obscure potentially smaller effects of environmental conditions in modulating viral prevalence.

Contrary to honey bees, we found that viral prevalence in wild bees was related to environmental conditions, whereby intermediate temperatures and intermediate precipitation of the warmest and driest quarters led to the highest viral prevalence in wild bees.

The impact of climatic conditions, in particular those related to heat and drought stress (precipitation in the driest and in the warmest quarter, temperature in the driest quarter), was consistent across all three viruses. For these environmental conditions, viral prevalence in wild bees was highest in the most moderate (intermediate) conditions and lowest at the extreme ends. Here one might argue that these extremes of both precipitation in the warmest and driest quarter as well as temperature in the driest quarter are likely suboptimal for most pollinator species as well as their floral resources. This is either due to a lack of rain-free days for foraging or drought or a combination of drought and heat, which may have a negative impact on the availability and attractiveness of floral resources^44,47^. These suboptimal conditions can induce physiological and nutritional stress, which can either result in reduced host populations^73,74^ and hence lower viral transmission and prevalence or result in an increased virulence of viral infections due to stressed hosts^63^. Malnutrition has been shown to negatively affect a host’s defense system^75^, which can increase virus-induced mortality^76^, as sown for Slow bee paralysis virus and DWV infections in bumble bees^63,77^, resulting in lower viral prevalence^78,79^. In the light of climate change, which has been shown to impact host–pathogen interaction^80,81^, we anticipate that these extremes in both temperature and precipitation will increase^82,83^. This, together with the ongoing loss of suitable naturally occurring floral resources^84^, can further increase the nutritional stress on wild bees, which may amplify pathogen stress^85^, and hence warrants further research. Besides their direct effect on hosts, these environmental conditions may also affect the viruses themselves. Heat, UV-exposure and drought may impact virus viability on the flowers, and increased rainfall may increase wash-off or dilute the virus below its minimal infection dose, leading to lower viral transmission at flowers.

We found only weak support for environmental modulation of the relationship between viral prevalence in honey bees and wild bees. Here the link between viral prevalence in honey bees and wild bees increased with the length of the vegetation period (Supplementary Fig. 2b), which may be attributed to prolonged foraging activity, facilitating transmission across a longer period of time together with the increase in viral prevalence in honey bee colonies as the foraging season progresses^14,86^.

Overall, our study shows at a continental scale that wild bee viral prevalence is affected by both biotic factors, namely viral prevalence in sympatric honey bees, and abiotic factors, specifically climatic conditions. The role of managed honey bees as an impact on wild bee viral prevalence has been identified before on a smaller scale, yet the link with climate has to our knowledge not been addressed at a continental scale. Temperature and precipitation extremes will likely continue to increase in the coming years due to climate change, and pathogens can exert negative effects when their interaction dynamics change^85^. Understanding pathogen prevalence in an environmental context, especially that of threatened species, can improve conservation strategies and hence deserves more attention.

## Methods

The bee sampling, identification, processing and virus detection protocols were performed as described in Miranda et al.^87^, and described in short here below. Sample collection, identification, processing, and cDNA synthesis was all done by the local partner. The virus assays were conducted by the partners in Belgium, France, Germany, Sweden, and Switzerland.

### Bee collection

Foraging adults were collected at 12 sites, in 11 countries across a climatic gradient in Europe (Fig. 1). The field sampling was performed according to a standardized sampling protocol^87^, where each partner sampled in their respective country. At each site 30 honey bees and 30 wild bees were collected in the order they were encountered as well an additional 15 specimens of the most common wild bee. The presence of wild honey bees is close to non-existing in Europe, with the exception of a few records. It is assumed that these wild colonies disappeared due to the introduced parasitic mite *Varroa destructor* and its viral vectoring capacity^88,89^. It is therefore reasonable to presume that nearly all the caught honey bees in this study originate from managed colonies and not from wild ones. At each site, all specimens were collected on the same day and in the same flower-rich area (ca. 100 m^2^) and then stored individually on ice after capture. Upon return to the laboratory, all samples were stored immediately at − 80 °C until further analysis. Sampling date varied between sites as bees were collected between April and September on a day with high foraging activity.

### RNA extraction from individual bees and cDNA production

For RNA extraction of the bees, we used the protocol described by de Miranda et al.^87^. For each bee we only used the abdomen, which was dissected sterile and crushed in a TBS-buffer (50 mM TRIS.HCl PH 7.4, 150 mM NaCl). The volume of TBS buffer used differed depending on the size of the abdomen, i.e., 800 µl for bumble bee sized abdomens, 500 µl for honey bee sized abdomens and 200 µl for small sweat bee sized abdomens. The homogenate was centrifuged to spin down the exoskeleton then 100 µl of supernatant was mixed with 350 µl RLT buffer supplemented with 1% beta-mercaptoethanol. RNA extraction was done using the Qiagen Plant RNeasy Plant kit (Qiagen) according to manufacturer’s protocol and was eluted with 50 µl (for large bees) or 30 µl (for small bees) nuclease free water. RNA concentrations were measured using NanoDrop and adjusted to 100 ng/µl using nuclease free water. cDNA was prepared using random hexamer primers and 1 µg RNA and cDNA kit (#K1612, ThermoFisher Scientific, Waltham, Massachusetts, United States of America) containing M-MLV reverse transcriptase and RNAse inhibitor in a 20 µl volume according to the manufacturer’s protocol.

### Virus screening

All bees were screened for three common bee virus targets, namely the AKI-virus complex (acute bee paralysis virus, Kashmir bee virus and Israeli acute paralysis virus), deformed wing virus and slow bee paralysis virus.

These viruses were chosen based on their wide distribution, relative high prevalence, and broad host range where they are detected in both wild bees and honey bees, which was a perquisite to test our hypothesis on the impact of honey bees as a biotic factor on wild bee viral prevalence.

Each PCR reaction contained 2 µl of diluted (1/10) cDNA and 18 µl qPCR mixture, containing 0.2 µl of both forward and reverse primers. For primer sequences, see Supplementary Table 2. Although the used primers are broad-range, capable of detecting several viral strains and species within a complex, we are aware that they are not flawless and may have missed some viral strains/variants due to the high mutation and recombination rates of these RNA-viruses.

### Statistical analysis

Viral prevalence was calculated for honey bees and wild bees, aggregated into two taxonomic groups, bumble bees and solitary bees, as the fraction of specimens scored positive in virus detection. We used a Cq threshold of 35 to define positive viral detection (Cq < 35) per species group. As we report the detection of viruses and classify a bee as positive if Cq values are below the set threshold, we are aware that we cannot state that positive bees are truly infected. This would require the detection of replicating virus (e.g. by detecting the negative strand of these positive strand RNA viruses), which we have not done. All analysis were performed using R (version 4.0.4)^51^ and the following packages (rgdal, raster, sp, dplyr, Hmisc, glmmTMB, MuMIn, ggplot2, effects)^90–98^.

### Explanatory variables

We selected an initial set of the following nine bioclimatic variables covering relevant temperature and precipitation conditions: annual mean temperature (BIO1), temperature seasonality (BIO4), mean temperature of driest quarter (BIO9), mean temperature of warmest quarter (BIO10), mean temperature of coldest quarter (BIO11), annual precipitation (BIO12), precipitation seasonality (BIO15), precipitation of driest quarter (BIO17), and precipitation of warmest quarter (BIO18).

Bioclimatic variables were obtained from CHELSA (v1.2) at a resolution of 30 arc seconds (about 1 km) averaged across the years 1979–2013^99,100^. To analyze the impact of activity period and time of sampling in the respective sampling year, we extracted data on the start and length of the vegetation period for each site as a proxy for the time of flower availability. Calculation of the vegetation period was based on the Normalized Difference Vegetation Index (NDVI) and provided by the Vegetation Index and Phenology Lab at a 3 arc min (about 5.6 km) resolution (https://vip.arizona.edu/). The actual sampling date was related to the start of the vegetation period per site (number of days since start of the vegetation period) and is referred to as ‘sampling phenology’.

All variables were tested for collinearity with a hierarchical cluster analysis based on pairwise Spearman rank correlation coefficients using UPGMA (unweighted pair-group method with arithmetic averages) agglomeration. Only non-collinear variables were retained (Spearman r < 0.7). Selection from clusters of collinear variables was based on ecological relevance (e.g. mean temperature of the warmest quarter was selected instead of annual mean temperature). After selection, the following six variables remained: mean temperature of driest quarter, mean temperature of warmest quarter, precipitation of driest quarter, precipitation of warmest quarter, length of vegetation period, and sampling phenology.

### Model development

To test the impact of climate and phenology on honey bee viral prevalence, we used a generalized linear mixed effects model (GLMM) with a binomial error structure (prevalence between 0 and 1) and a logit link, weighted by sample size. Climatic variables were included with their linear and quadratic terms. All three viruses were considered in one model to test for differential responses among the viruses, i.e., interaction between virus type and environmental variable. As crossed random effects, we considered virus type to avoid pseudo-replication, assay laboratory to account for potential systematic differences among the analyzing laboratories and an observer term to address overdispersion where necessary.

To test the relationship between viral prevalence in wild bees and honey bees and the, potentially modulating, impact of environmental variables, we used the same approach as described above but added main effects, two-way and three-way interactions of viral prevalence in honey bees, environment, and virus type. Bumble bees and solitary bees were considered in one model. Group-specific responses to viral prevalence in honey bees were initially tested with interaction effects but without environmental variables to avoid model overfitting. No interaction was evident and thus it was excluded from subsequent analyses. We also added bee taxon (bumble bees, solitary bees) as an additional random effect.

To avoid model overfitting, we limited the number of fixed effects and each environmental variable was tested separately while keeping sampling phenology as a covariate to control for potential differences in viral prevalence due to differences in the time of sampling across the year. We used a multi-model inference approach based on the Akaike Information Criterion corrected for small sample sizes (AICc)^101^ for model simplification. We considered models with a delta AICc lower than 2, towards the best model, having strong support and models with delta AICc between 2 and 4 as having weaker support. We disregarded a set of models for a particular environmental variable if the intercept-only model was within the respective subset (set of models with delta AICc < 2 and set of models with delta 2 < AICc < 4).

## Supplementary Information

Supplementary Information 1.

Supplementary Information 2.

## Data Availability

The dataset is included as a supplementary file.
