# Supplementary material for: Honey bees and climate explain viral prevalence in wild bee communities on a continental scale
Source: Sci Rep. 2022 Feb 3;12:1904. doi: 10.1038/s41598-022-05603-2 (PMC8814194; doi:10.1038/s41598-022-05603-2)
Supplement: Supplementary file 2 — Supplementary Information 2. [file 41598_2022_5603_MOESM2_ESM.pdf]

Supplementary Information

Supplementary Table 1: Multi-model inference results for separate analyses of the impacts of climatic and vegetation phenological conditions on viral prevalence in honey bees.

Intercept	Env	Env^2	Phen	Virus	Env:Virus	AICc	Delta	Weight
Temperature driest quarter								
-3.45				+		169.09	0.00	0.55
-3.18		-0.23		+		172.05	2.95	0.13
-3.52	0.23			+		172.08	2.98	0.12
-3.79			< 0.01	+		172.09	2.99	0.12
-2.28						172.88	3.79	0.08
Temperature warmest quarter								
-3.45				+		169.09	0.00	0.55
-3.79			< 0.01			172.09	3.01	0.12
-3.66		0.17		+		172.12	3.02	0.12
-3.42	-0.05			+		172.19	3.09	0.12
-2.28						172.88	3.79	0.08
Precipitation driest quarter								
-3.45				+		169.09	0.00	0.53
-2.93		-0.51		+		171.63	2.54	0.15
-3.79			< 0.01	+		172.09	3.01	0.12
-3.45	-0.11			+		172.13	3.04	0.12
-2.28						172.88	3.79	0.08
Precipitation warmest quarter								
-3.45				+		169.09	0.00	0.54
-3.03		-0.32		+		171.60	2.50	0.15
-3.79			< 0.01	+		172.09	3.01	0.12
-3.45	0.03			+		172.19	3.10	0.11
-2.28						172.88	3.79	0.08
Length of vegetation period								
-3.45				+		169.09	0.00	0.54
-3.57		0.18		+		171.92	2.82	0.13
-3.43	0.18			+		172.02	2.93	0.13
-3.79			< 0.01	+		172.09	3.01	0.12
-2.28						172.88	3.79	0.08

Env, linear term of respective environmental variable; Env^2, squared term of respective environmental variable; Phen, sampling phenology; Virus, virus species; colon indicates interaction terms. Numbers are coefficient estimates of continuous variables; '+' indicates relevance of categorical variables. AICc, Akaike information criterion corrected for small sample size; Delta; delta AICc; Weight, Akaike weight. Intercept only model indicated with grey background.

Supplementary Table 2: qPCR primers used for virus detection. From left to right column: the target virus, primer names, primer sequence, size of the fragment and literature reference of the primer

TARGET	PRIMERS	SEQUENCE '5-'3	SIZE (bp)	Ref
AKI-complex	KIABPV-F6648 (F)	CCTTTCATGATGTGGAAAC	98	[1]
	KIABPV-B6707 (R)	CTGAATAATACTGTGCGTATC		
SBPV	SBPV-F3177 (F)	GYGCTTTAGTTCAATTRCC	226	[2]
	SBPV-B3363 (R)	ATTATRGGACGTGARAATATAC		
DWV	DWV-F8688 (F)	GGTAAGCGATGGTTGTTTG	143	[1]
	DWV-B8794 (R)	CCGTGAATATAGTGTGAGG		

Supplementary Figure 1: Prevalence of three viruses: AKI-complex (Acute bee paralysis virus, Kashmir bee virus, Israeli acute paralysis virus), Deformed wing virus (DWV), Slow bee paralysis virus (SBPV) in honey bees, bumble bees and solitary bees across 12 sites in 11 European countries (ISO2 country code). Countries are ordered with increasing prevalence of DWV in honey bees.

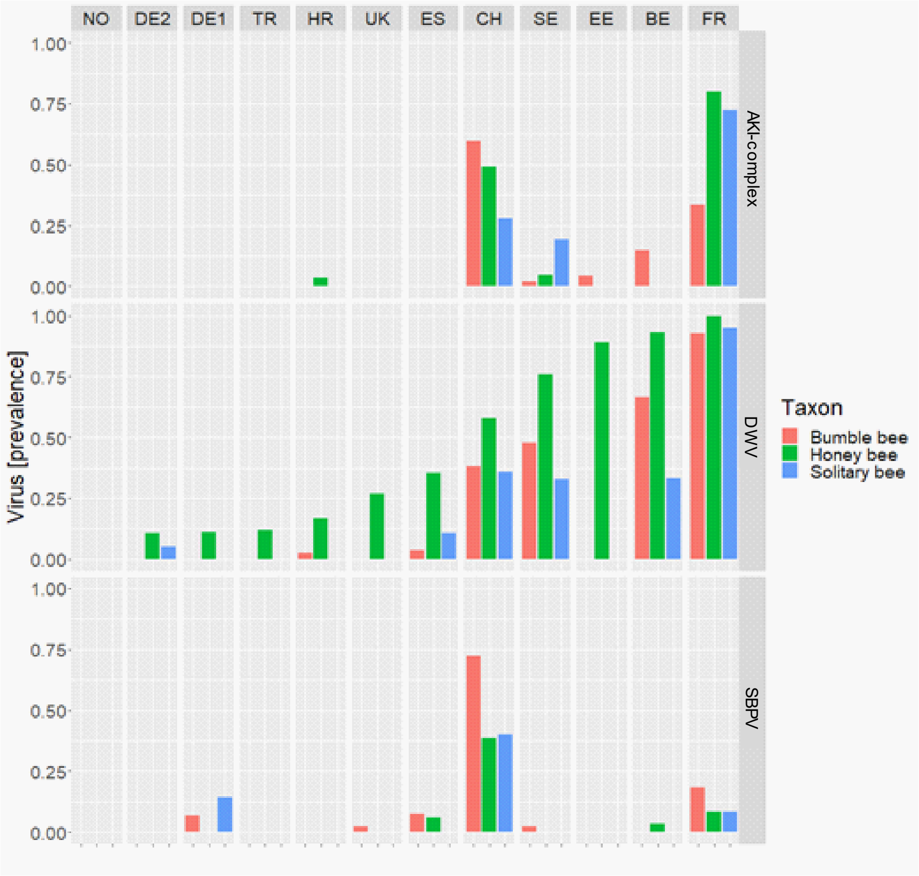

Sample size per sampling site (from left column to right), BB: number of bumble bees; SB: number of solitary bees; HB: number of honey bees: NO[BB:40;HB:30]; DE2[BB:23;SB:19;HB:28]; DE1[BB:15;SB:14;HB:27]; TR[BB:27;SB:19;HB:34]; HR[BB:37;SB:5;HB:30]; UK[BB:45;HB:30]; ES[BB:27;SB:28;HB:34]; CH[BB:90;SB:27;HB:60]; SE[BB:94;SB:73;HB:87]; EE[BB:24;HB:46]; BE[BB:27;SB:12;HB:29]; FR[BB:27 ;SB:59 ; HB:60]

Supplementary Figure 2: Relationship between viral prevalence in wild bees and honey bees depending and on the temperature of the warmest quarter under cool (18 °C) and warm (22.4 °C) conditions (A) and on the length of the vegetation period depicted for 260, 265, 270, and 300 days (B).

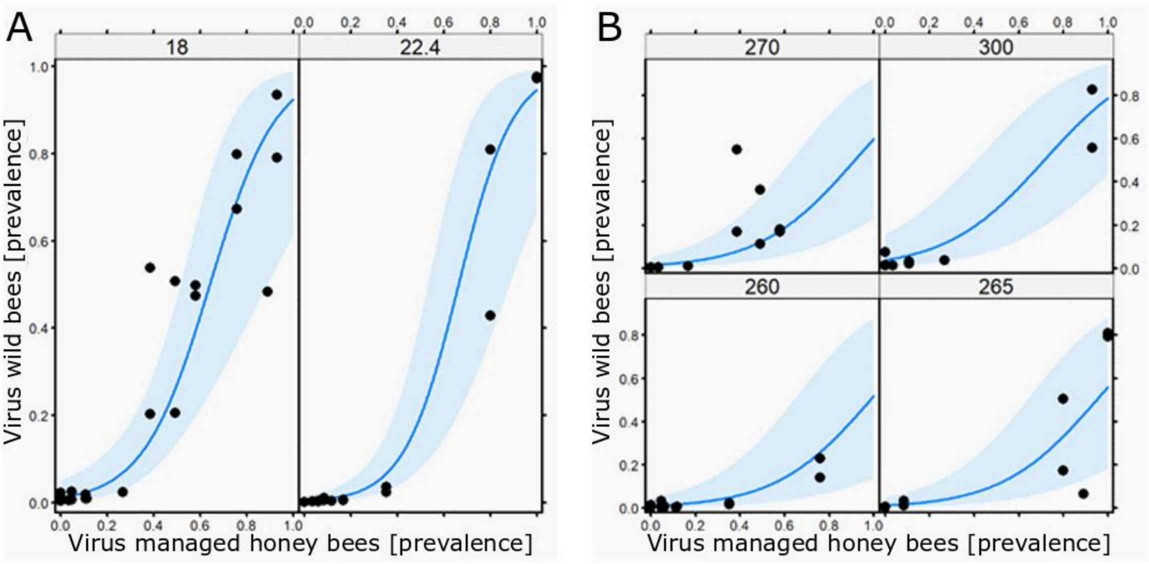

Supplementary Figure 3: Relationship between viral prevalence in wild bees and mean temperature of the warmest quarter split by virus species.

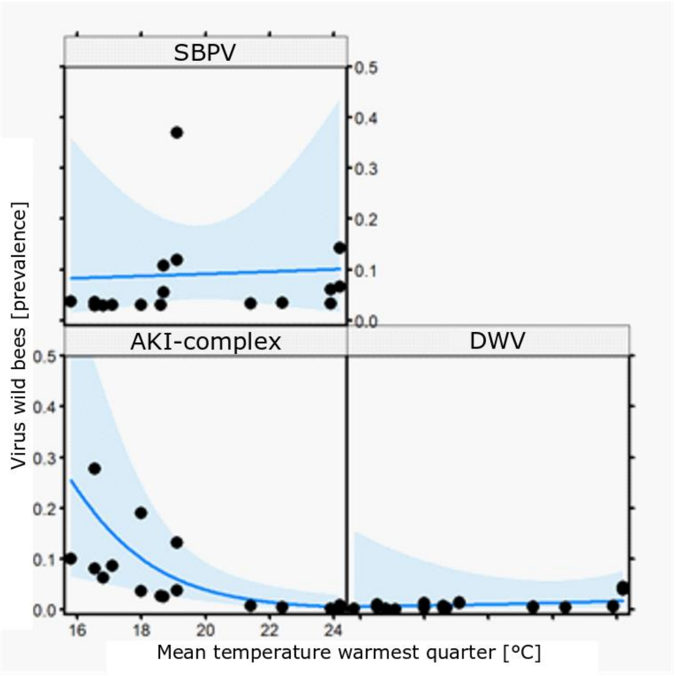

References

1. Mondet, F., de Miranda, J. R., Kretzschmar, A., Le Conte, Y. & Mercer, A. R. On the Front Line: Quantitative Virus Dynamics in Honeybee (Apis mellifera L.) Colonies along a New Expansion Front of the Parasite Varroa destructor. *PLoS Pathog.* **10**, e1004323 (2014).
2. de Miranda, J. R. *et al.* Genetic characterization of slow bee paralysis virus of the honeybee (Apis mellifera L.). *J. Gen. Virol.* **91**, 2524–30 (2010).
